# Effect of THz-bandwidth incoherent laser radiation on bulk damage in potassium dihydrogen phosphate crystals

**DOI:** 10.1038/s41598-024-55732-z

**Published:** 2024-03-04

**Authors:** Douglas Broege, Michael Spilatro, Guillaume Duchateau, Christophe Dorrer, Stavros G. Demos

**Affiliations:** 1 Laboratory for Laser Energetics, University of Rochester, 250 East River Road, Rochester, NY 14623-1299 USA; 2 CEA-CESTA, 15 Avenue Des Sablières, CS60001, 33116 Le Barp Cedex, France

**Keywords:** Energy science and technology, Engineering, Materials science, Optics and photonics, Physics

## Abstract

The laser-damage performance characteristics of potassium dihydrogen phosphate (KDP) samples under exposure to a distinctive broadband incoherent laser pulse are investigated. A laser system providing such pulses is intended to explore improved energy-coupling efficiency on the target in direct-drive inertial confinement fusion experiments and provides incoherent bandwidths as large as 10 THz in a nanosecond pulse. A consequence of this bandwidth is very rapid fluctuations in intensity capable of reaching maxima much larger than the average intensity within the pulse. A custom damage-test station has been built to perform measurements with broadband incoherent pulses in order to determine what effect these fast and high-intensity oscillations have on laser damage. A set of experiments under different bandwidth and beam configurations shows the effect to be minimal when probing bulk damage in KDP. Modeling indicates this behavior is supported by long electron-relaxation times compared to the source-field fluctuations, following excitation of individual electrons in the conduction band. The results help better understand the laser-induced–damage mechanisms in KDP, and its ability to operate in broadband temporally incoherent high-energy lasers that may be particularly suitable for future laser-fusion energy systems.

For decades, the potential for inertial confinement fusion (ICF) as a viable energy source has been explored with consistent incremental progress. The most-recent announcement that ignition has been achieved at the National Ignition Facility^1^ has bolstered interest not only in ICF, but also in technologies necessary to enable inertial fusion energy. Laser direct drive^2^, a process where the fusion target is directly irradiated by the laser beams, could provide optimal energy-coupling efficiency if several parametric instabilities that occur between the driving laser light and the formed plasma can be addressed^3,4^. These interactions limit the amount of energy that can be deposited on target and significantly limit the efficiency of the process. Simulation results suggest that these interactions can be significantly suppressed by utilizing an incoherent broadband driving field^5–7^. Recent efforts to realize such a source based on novel optical parametric amplifier (OPA) technology, which amplify fluorescence-based seed light, producing nanosecond pulses with an incoherent bandwidth of 10 THz have been demonstrated^8–10^. Although lasers of this type produce relatively long pulses, their incoherent spectrum results in intensity fluctuations that occur on time scales as short as 100 fs and reach peak intensities much larger than their nominal intensity (ratio of energy to overall pulse duration), in contrast to currently operating high-energy laser systems..

Given that laser-damage mechanisms require the coupling of energy from the laser beam into the material, such processes can occur in large band-gap dielectric materials via nonlinear absorption. With this in mind, there is good reason for concern that such fluctuations in intensity can cause significant changes in damage probability in optical materials. For this reason, previous investigations have been carried out in silica utilizing *Q*-switched lasers with injection seeding capability. These lasers produce a temporally smooth pulse when seeded, which can be compared to unseeded operation, where a number of longitudinal modes interfere to create a temporally modulated pulse with increased bandwidth^11,12^. One such study by Diaz et al. has demonstrated an increase in damage density with increased bandwidth that is attributed to the higher-order multiphoton absorption in the infrared. In contrast, a reduction in damage density was observed with ultraviolet pulses, where lower-order multiphoton absorption is required^12^. Additional work by Bouyer et al., which was designed to more-efficiently isolate the effects of modulation frequency and amplitude on damage in silica^13^, was performed using amplitude-modulated nanosecond pulses, and reported similar results. These fundamental works cover the effects of GHz bandwidths accessible by modern electronics or associated with mode beating in *Q*-switched lasers, and are limited to silica. Additionally, the amplitude of the associated intensity fluctuations is on the scale of 2 × the average. We present here the first study aiming to understand the potential risks of THz-scale fluctuations from broadband incoherent laser radiation on optical materials, and in particular, in the nonlinear material KDP, which is a key candidate material for the generation of such laser pulse types in large aperture formats.

Owing to their ability to grow to large sizes, KDP and its deuterated analog DKDP are uniquely suited for use in large-aperture ICF-class lasers for a number of applications, including distributed phase plates for beam smoothing^14^, and nonlinear crystals for second- and third-harmonic generation^15^. As a result, these materials have been the subject of numerous studies covering laser-induced–damage thresholds (LIDT)^16,17^, laser conditioning^18^, and modeling of damage initiation^19^ that can be used to interpret the findings of the present work.

In this work, the laser-damage performance characteristics of potassium dihydrogen phosphate samples under exposure to a broadband incoherent laser pulse are investigated. The experimental section of the paper describes the dual-beam laser source and damage-test station designed to help understand how the distinct features of the temporally fluctuating pulses affect laser damage. The analysis section covers data processing, the calculation of relevant quantities such as damage probability and damage density, and a discussion of experimental results. The final section utilizes an established model that enables the estimation of the transient free-electron density in KDP during laser irradiation with THz-scale fluctuations and correlates to parameters relevant to laser-damage initiation.

## Experiment

**Figure 1:**
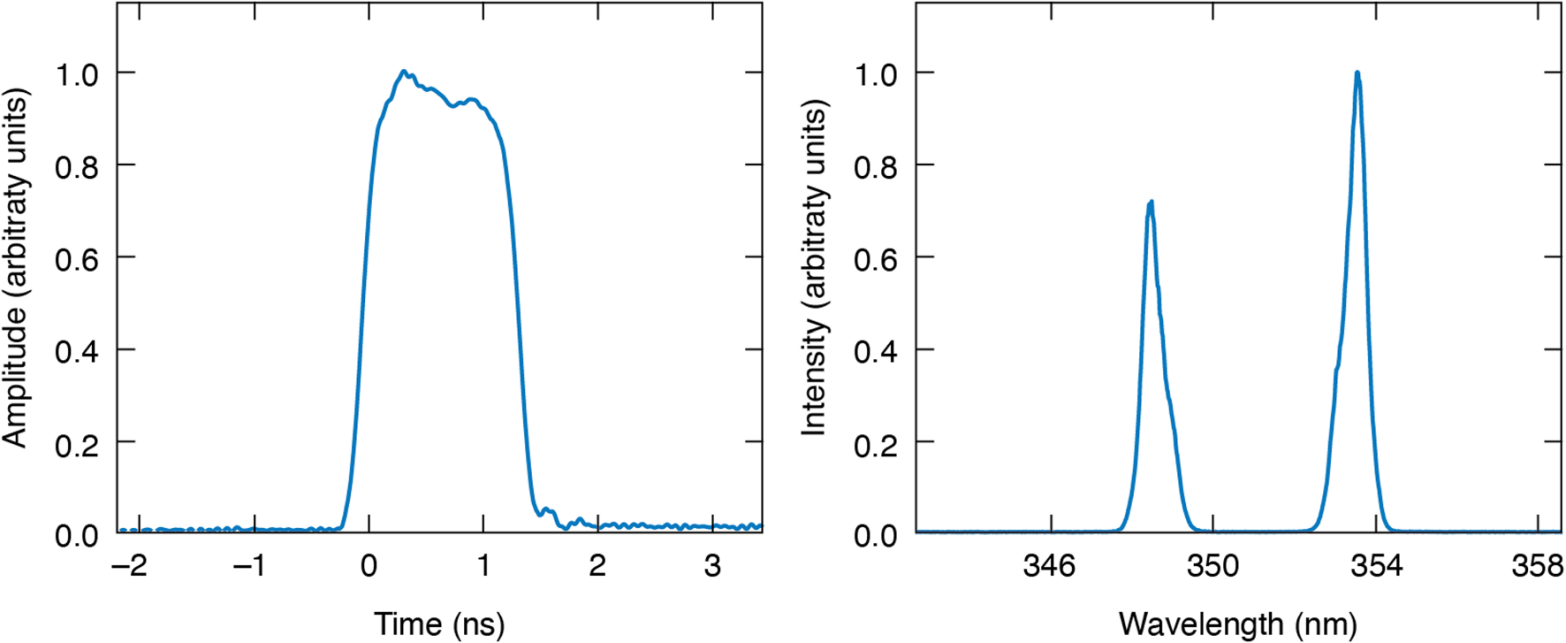
Representative pulse shape measured with a narrowband seed (left), and spectrum of the laser used for damage testing with a broadband incoherent seed (right).

The laser source used in this experiment is OPA-based and is seeded by either a narrowband fiber laser or a broadband fluorescence source, depending on the desired output. The seed pulse is electro-optically shaped and is amplified through one OPA stage, resulting in a signal–idler pair centered at 1030 nm and 1076 nm, respectively. Owing to the collinear nature of the OPA, these two beams are spatially overlapped and propagate in the same direction. They are combined with a 527-nm narrowband beam for broadband sum-frequency generation to produce a UV pulse having a spectrum with peaks centered at 348 nm and 354 nm. Further information on this system can be found in^9^. Figure 1 shows a measured UV pulse shape and spectrum representative of test conditions. When operating in a broadband configuration, each beam has a bandwidth of around 3 THz, which results in two different types of temporal fluctuation. The first is random, occurring on time scales as short as a picosecond, and is the consequence of the broadband incoherent spectrum of each beam. The second corresponds to beating between the signal and idler beams and is much more regular, with a period close to 100 fs. Since each of these fluctuation types may have their own effect on laser damage, we conducted a number of separate damage tests utilizing 1.5 ns pulses with different bandwidth and beam configurations: (1) a narrowband signal beam; (2) a broadband signal beam; (3) a narrowband idler; (4) a broadband idler; (5) a narrowband beam pair; and (6) a broadband beam pair.

**Figure 2:**
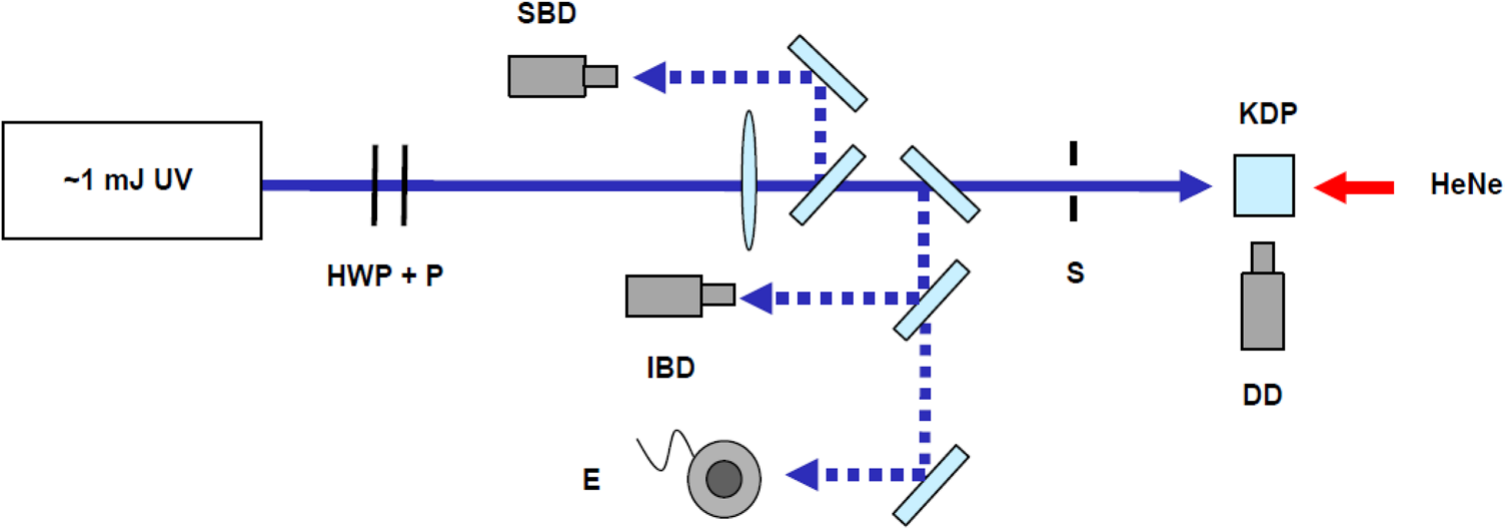
Experimental setup. Due to the wavelength-dependent response in cameras, separate beam diagnostics are necessary to accurately calculate fluence on shot in dual-beam tests. Both beams follow each diagnostic path, and are separated before the camera with spectral filters. HWP + P: half-wave plate and polarizer used for beam attenuation; SBD: signal beam diagnostic; IBD: idler beam diagnostic; E: energy meter; S: shutter; KDP: KDP sample on *x*–*y*–*z* translation stage, DD: 1 × bulk damage diagnostic camera; HeNe: HeNe laser used to illuminate the test volume.

A schematic of the experimental setup designed to test for bulk damage in KDP is shown in Fig. 2. With an available energy of 1 mJ, the damaging beam is focused to a spot with a diameter of 30 µm (1/e^2^) in order to reach the fluences necessary for testing. The Rayleigh range of this beam is shorter than the thickness of our samples, so the samples were positioned with their front surfaces at the beam waist. Detection of damage is performed in situ with background-subtracted images collected with a camera capable of imaging the entire laser-exposed length of the samples from the side, which is polished to facilitate observation. Illumination is provided with a HeNe laser counter-propagating along the same path as the damaging beam in order to isolate the effects of the damaging beam from other scattering points and nearby test sites. Acquisition from the beam and energy diagnostics is coordinated with the operation of a shutter with custom software. In the tests described here, a single shot is delivered to each volume tested, and a declaration of whether damage has occurred is made by the operator. This is then used to calculate the probability of damage as a function of fluence.

An important aspect of laser-damage testing is an accurate measurement of fluence on shot, which can be calculated using data from beam and energy diagnostics. With two significantly different wavelengths present, accuracy can be affected by the spectrally varying response of a camera. For this reason, two separate beam diagnostic cameras are used—one for each beam. Samples of both signal and idler beams are sent through each diagnostic arm and are spectrally filtered so each camera sees only one beam. Before each test, a calibration is performed using a single beam and no spectral filters to determine the absolute spatial offset between the two images. This information is used in the data-acquisition software, where the images are normalized and summed to provide an accurate profile for the composite beam. An assumption is made in the fluence calculation that the energy in the beam is divided equally between signal and idler, which is confirmed to roughly be the case upon startup of the laser.

An additional factor to consider in a multi-beam study of bulk damage with focusing beams is the potential for the two beams to have somewhat different focal parameters. Focal scans for each beam revealed that while the signal focuses at the front face of the crystal, the idler beam focuses nearly 3 mm further downstream in free space. This difference comes from the fact that in an OPA, the signal’s wavefront is defined by the seed, while the idler’s depends on the wavefront of the seed and the pump. In practice, a best attempt at matching the two is made, but there will always be some difference. The ultimate effect on bulk damage testing is that the idler beam reaches peak fluence beyond the front face of the crystal, exposing the bulk to a higher fluence than is reported by beam diagnostics. For this reason, a corrective factor is applied to all diagnostic-calculated fluences when the idler is present. This factor is determined by using focal scan data to calculate the peak fluence relative to the front face of the crystal, and is approximately 1.2 for idler-only tests, and 1.06 for dual-beam tests.

**Figure 3:**
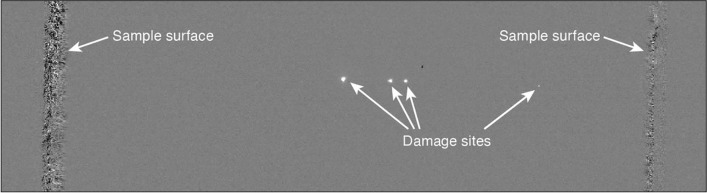
Background-subtracted image of sample after a damaging laser shot. In this example, four clearly identifiable scattering points appear along the path of the laser, indicating damage.

The data presented in this work were obtained from two nominally identical, commercially acquired KDP samples, aiming to minimize variation in damage performance arising from crystal quality. Both were 1 × 1 × 1.5 cm, (*x*–*y*–*z* cut) and polished on the four long faces to permit both laser transmission and viewing from the side. The damaging beam’s polarization was chosen to lie along the ordinary axis. An attempt was made to probe surface damage, but the fluences accessible to the test station were not able to consistently reach the surface damage threshold. For bulk damage, each test consisted of over 450 1-on-1 test sites arranged in a hexagonal array, with a spacing of 250 *µ*m between nearest adjacent sites. This site arrangement is chosen so damage from previously tested volumes is less likely to interfere with measurements, since the size of the formed damage sites is much smaller than the spatial separation between tested volumes. Figure 3 shows a typical background-subtracted image of the crystal recorded by the damage-detection imaging system. Damage morphology consists of anywhere between 1 and 20 clearly identifiable scattering points along the beam path that appear after a damaging laser shot. While no attempt was made to examine damage morphology via microscopy, the degree of damage observed through testing (i.e., the number of scattering points and their brightness) did not appear to vary significantly between different bandwidth conditions.

### Analysis and results

Analysis was performed by subtracting images captured by the damage-detection camera before and after laser exposure. Thresholding was used to identify damage and determine the number and location of scattering points within a tested volume. When compared to operator declarations of damage, only a few percent of the sites were flagged as mislabeled, the majority of which were the result of noise. Data outliers were revisited for direct inspection by the operator.

The calculation of damage probability for each test is performed by calculating the fraction of sites damaged within a 1-J/cm^2^ range around each data point. This approach is similar to the more-traditional binning recommended by the International Standard Organization, but avoids the variability that arises with an arbitrary choice of bin centers. This method still results in a nonzero damage probability for fluences lower than where damage is detected, so we also explicitly report the lowest fluence at which damage occurred in Table 1. Since the test range is higher than the design fluence of 2.5 J/cm^2^, a calculation of damage density is performed on the data and fit to a power law as a function of fluence, $D\left(F\right)={D}_{o}{\left(\frac{F}{{F}_{o}}\right)}^{\beta }$, following Ref.^20^. This fit is then used to extrapolate the expected damage density at operational fluences which are included in Table 1.

**Table 1:** Performance metrics for each test performed. Listed are the lowest fluence-inducing damage in each test; the coefficient *β*, resulting from fitting a calculation of damage density to a power law; and an estimated damage density at design fluence of 2.5 J/cm^2^ using the fit.

	Lowest damaging fluence	Power law fit coefficient *β*	Extrapolated damage density at 2.5 J/cm^2^ (cm^–3^)
Narrowband signal	9.2	6.9	11
Broadband signal	9.6	9.4	0.5
Narrowband idler	10.5	12.3	0.03
Broadband idler	10.0	7.7	6.4
Narrowband dual	8.3	7.7	13
Broadband dual	9.5	7.2	15

**Figure 4:**
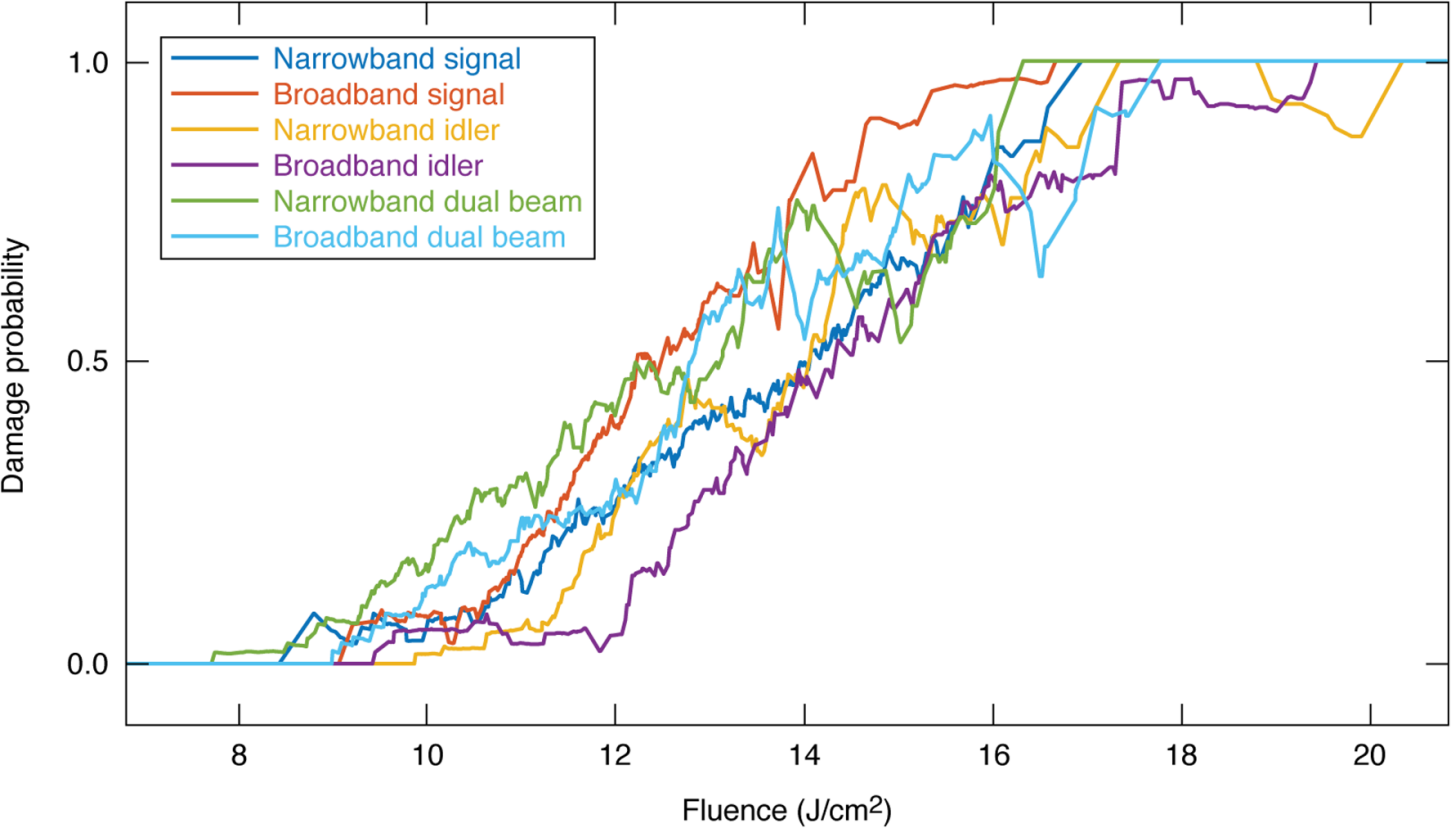
Damage probability for all six tests performed. Damage probability is calculated at each data point with a 1 J/cm^2^ wide bin. Data show that under these testing conditions, damage is probabilistic over a range of ~ 10 J/cm^2^, while variation between all tests is closer to ~ 2 J/cm^2^.

Figure 4 shows damage probability as a function of fluence for each of the six tests carried out. Because damage probability is expected to monotonically rise with fluence, fluctuations are an indication of statistical variability within a test. These fluctuations are larger near the boundaries since there are fewer test sites or damage events per bin. The range over which damage is probabilistic is generally the same for each test; additionally, the curves cross at a number of points, making the relative performance between bandwidth settings dependent on metric. A comparison between narrowband and broadband configurations shows a modest decrease in damage probability for the broadband case when the signal beam is used for testing, but the opposite is seen with the idler. The magnitude of these differences is smaller than the overall spread in test results, which is largely bounded by a pair of tests with the same bandwidth setting (broadband signal and broadband idler tests). This implies that the differences in results between broadband and narrowband settings are likely statistical, and if broadband radiation has an effect on laser damage, it is smaller than can be resolved with these tests.

In order to determine if laser damage is affected by the faster oscillations associated with beating between the two separate beams, comparisons can be made between tests utilizing either one or both beams, but with the same bandwidth setting. In the case of narrowband tests, a dual-beam configuration shows a somewhat increased damage probability over signal-only tests, while the opposite is true with broadband tests. When comparing idler-only tests to dual-beam tests, there is a moderate increase in damage probability for the dual-beam tests regardless of bandwidth configuration. The magnitude of this difference is generally smaller than those seen when comparing bandwidth configurations.

In summary, while some differences between tests exist, the results as a whole do not show any significant trends when varying test conditions such as bandwidth or the presence of a secondary beam. The implication then is that if an effect exists, it is smaller than these tests can resolve. This is clearest when observing that one of the larger differences seen between two tests exists between the signal and idler tests using a broadband seed, where the only difference is central wavelength. While the difference is consistent with the higher photon energy being more likely to damage, the wavelength dependence of laser damage in KDP has been thoroughly documented^16^, and cannot be solely responsible for the difference. It follows then that variation between the power-law coefficients determined by fitting to this data listed in Table 1 lies within experimental error. Assuming that the cumulative density of defects follows a power law as a function of fluence, we conclude that damage density at the intended operational fluence of this novel laser will be of the order of 10^–2^ to 10^1^ cm^–3^.

It is worth noting that there are a number of systematic aspects of this study that can affect the damage measurement. The first and most significant is the beam size. The spot size on target of 30 µm was chosen to achieve a reasonable testing fluence range with the available energy and achromatic optics. With defect-driven damage, a smaller spot size results in more probabilistic damage over a larger range of fluences, requiring greater statistics. The second factor is beam overlap in dual-beam tests. While offset between beam spots on different cameras is precisely established at the beginning of each test, mechanical drifts and minor changes in beam position from changing camera filters can occur, and also contribute to miscalculation in the composite beam profile. Finally, as described above, wavefront differences between the two converging beams can lead to different peak fluences in the bulk than reported by diagnostics. This is addressed with corrective factors derived from focal scans in free space, but they do not represent a direct measurement of the intensity distribution inside the samples.

### Modeling

In order to obtain a better understanding of the physical processes involved that lead to the observed minimal influence of the LIDT on THz-scale beam-intensity fluctuations, we have performed numerical simulations based on the model of laser-induced damage in KDP developed in^19^. Briefly, this model is based on a local increase in absorption due to defects that subsequently induce heating of the material and cascade the generation of new stoichiometric defects. These defects modify the electronic band structure by introducing electronic states located within the band gap supporting electron excitation via sequential single-photon absorptions bridging adjacent states, leading to ionization (i.e., the production of free electrons). The excited electrons are also allowed to recombine to the valence band through emission of phonons within a characteristic time scale of the order of 1 ps, resulting in energy deposition within the lattice. The density of free electrons has been shown to be a good criterion for determining damage-inducing fluence, and has been used to explain experimentally observed laser-induced damage in KDP crystals with narrowband nanosecond pulses, which is more commonplace in this type of testing^21^.

**Figure 5:**
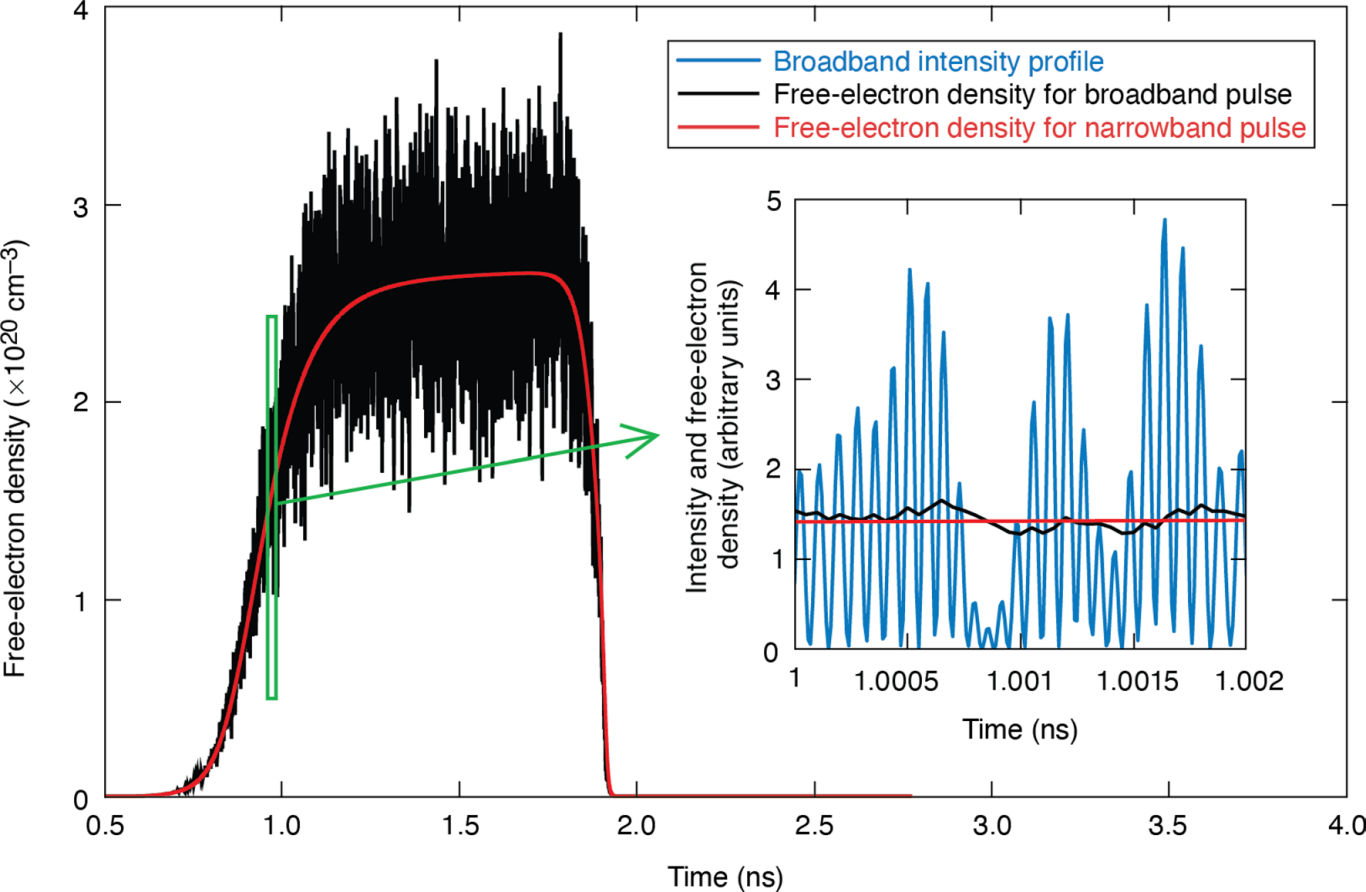
Temporal evolution of the laser-induced free-electron density in the center of an absorbing defect for a narrowband laser pulse (red curve) and an incoherent broadband laser pulse from two beams (black curve). The inset shows a zoom in the region *t* = 1 ns to show the electron-density variations more clearly. The associated laser-intensity profile is also provided (blue curve).

Numerical simulations based on this model have been carried out using laser pulse shapes exhibiting the same set of spectral properties as those used in the present experiment. Theoretical damage thresholds using all beam configurations used in the experiments vary within a range of a few percent (results not shown), which is consistent with experimental observations. To explain this behavior, a characteristic temporal evolution of the free-electron density induced by a 1.5-ns laser pulse with a fluence of 7 J/cm^2^ is shown in Fig. 5 for both broadband (broadband-seeded beam pair) and narrowband (narrowband-seeded signal beam) spectra. In the case where a narrowband spectrum is used, the temporal evolution of the intensity is smooth, leading to a smooth rise in free-electron density. In the broadband case, the laser beam intensity fluctuates with respect to time, leading to fluctuations of the electron density. There are two aspects of how the electron density varies in the broadband case that should be considered: (1) the average value of the electron density is close to the evolution of the density induced by a narrowband pulse, and (2) the relative amplitude of the electron-density fluctuations is much smaller than that of the intensity (see inset of Fig. 5). The time window is chosen at 1 ns, following^19^, because critical plasma density takes place early in the pulse. These observations can be attributed to the fact that the interaction consists of single-photon absorptions, leading to a linear response for each excitation. Second, the relaxation time scales of excited electrons are often longer than the characteristic period of fluctuation of the intensity (the shortest electron-relaxation time scale is 1 ps and the laser fluctuation period can be as fast as 100 fs). The electron system is therefore not able to follow the fast intensity dynamics; the density evolution is an average of intensity fluctuations as shown in the inset of Fig. 5. As a consequence, the amplitude of electron-density fluctuations is relatively small, explaining the fact that damage probability does not strongly depend on the pulse spectrum within the THz-scale beam-intensity fluctuation conditions.

## Conclusion

We have presented for the first time, to the best of our knowledge, damage-test measurements performed with THz-scale incoherent bandwidths projected to be capable of mitigating laser–plasma instabilities on target in laser-fusion direct-drive conditions. The results show that in KDP crystals, radiation of this type has little to no effect on bulk-damage probability compared with narrowband nanosecond pulses. Since these fields contain much higher peak field strengths than the nanosecond pulses conventionally used for damage testing in the context of high-energy laser systems, this suggests that the physical processes responsible for damage are not dominated by direct nonlinear excitation. Instead, modeling indicates that the effective relaxation time scale of electrons in this system, which occur on a picosecond time scale, act to average out the effects of intensity fluctuation that occur on faster time scales. The additional benefit of this work is that the results serve to further validate an established model describing damage mechanisms in KDP, making it a valuable tool in a field in which the majority of calculations related to damage initiation are based on silica.

From a laser-design standpoint, these results are valuable as they demonstrate the feasibility of utilization of a key optical material that enabled the generation of such broadband pulses. Further work is underway to support the development of other laser materials and coatings required in such laser systems. It is also noteworthy that since previous studies have shown an interesting dependence of LIDT on incoherent bandwidth on the GHz scale in fused silica^11^, it is possible that testing with various bandwidths could provide valuable information on time scales relevant to a particular damage mechanism and assist in the development of robust theoretical models of laser damage.

## Data Availability

Data is available on reasonable request from the contact author at dbro@lle.rochester.edu.
